# DynaVenn: web-based computation of the most significant overlap between ordered sets

**DOI:** 10.1186/s12859-019-3320-5

**Published:** 2019-12-30

**Authors:** Jérémy Amand, Tobias Fehlmann, Christina Backes, Andreas Keller

**Affiliations:** Chair for Clinical Bioinformatics, Saarland University, Saarbrücken, 66123 DE Germany

**Keywords:** Venn diagrams, Web server, Hypergeometric test, List overlap

## Abstract

**Background:**

In many research disciplines, ordered lists are compared. One example is to compare a subset of all significant genes or proteins in a primary study to those in a replication study. Often, the top of the lists are compared using Venn diagrams, ore more precisely Euler diagrams (set diagrams showing logical relations between a finite collection of different sets). If different cohort sizes, different techniques or algorithms for evaluation were applied, a direct comparison of significant genes with a fixed threshold can however be misleading and approaches comparing lists would be more appropriate.

**Results:**

We developed *DynaVenn*, a web-based tool that incrementally creates all possible subsets from two or three ordered lists and computes for each combination a p-value for the overlap. Respectively, dynamic Venn diagrams are generated as graphical representations. Additionally an animation is generated showing how the most significant overlap is reached by backtracking. We demonstrate the improved performance of *DynaVenn* over an arbitrary cut-off approach on an Alzheimer’s Disease biomarker set.

**Conclusion:**

*DynaVenn* combines the calculation of the most significant overlap of different cohorts with an intuitive visualization of the results. It is freely available as a web service at http://www.ccb.uni-saarland.de/dynavenn.

## Background

A frequent task for life science and biomedical researchers is to compare ordered lists. A prominent example is the comparison of genes, proteins or other biomarkers from a primary to a replication study. Often, the most significant markers are selected and the respective sets from the two studies are compared. Venn diagrams, or more generally Euler diagrams, offer themselves for visualizing the overlap between two or more respective sets. In addition to natural sciences, life sciences and medical applications they are also applied also in other research disciplines such as social sciences over economics studies [1–4]. A variety of online tools, stand-alone software programs or packages for toolboxes such as R have been implemented. Given two or more input sets, these tools compute either standard Venn diagrams or area proportional Venn diagrams, where the overlap between the sets corresponds to the area of the respective intersection in the Venn diagram. Frequently used tools among others include Venny (http://bioinfogp.cnb.csic.es/tools/venny/), BioVenn [5], the R Package VennDiagram, or jvenn [6]. All these tools excel by their ease of use and convenient representation of the results as planar Venn diagrams. To compute the significance of the overlap of two or more sets, the hypergeometric distribution can be applied. As mentioned, the sets to be compared are often the top part of an ordered list, e.g. the most significant genes for one disease that are compared between two studies. Here, fixed cut-off values are frequently applied, e.g. all genes with p-value below 0.05 are selected or all genes that are at least two-fold up-regulated. In some cases, e.g. for bimodal distributions, the selection of the threshold can be done from the distribution of the input data. Often, the selection of the most appropriate cut-off values may be challenging. First, it can be argued which cut-off is applied. Second, the cut-off for the two studies to be compared may have to be selected differently. The *p*-value for genes, e.g. computed by t-test or other hypothesis tests varies also with the cohort size, the used experimental technique and other factors. As an alternative, a fixed number of genes independent of the *p*-value is often considered, e.g. the top-20 dys-regulated genes in the two studies to be compared. If two ordered lists are available metrics for the comparison of such lists represent a valid alternative. Examples include Kendall’s Tau or Spearman’s Footrule that can be applied to compare the complete lists. All these strategies have their different strengths and weaknesses. As a flexible tool that does not rely on fixed thresholds and cut-offs we developed *DynaVenn*, a solution that computes the optimal overlap between two or three sets from ordered lists. Notably, a similar approach is followed by the R package *GeneOverlap* [7], performing Fisher’s exact test. This tool however uses a fixed threshold by taking the entire input lists and does not produce an interactive visualization. The genomic background is set to all genes measured in Chromatin ImmunoPrecipitation DNA-Sequencing (ChIP-Seq) or RNASeq while *DynaVenn* uses the genes measured in all the input sets. The web-based solution is freely available at http://www.ccb.uni-saarland.de/dynavenn.

## Methods & Implementation

### Basic concept

*DynaVenn* makes use of the general concept and intuitive graphical representation of area proportional Venn diagrams. For two or three ordered lists of items (*A*, *B*, *C*) all possible Venn diagrams are calculated by considering the top *l*, *m* and in case of three lists *n* items as sets. In the case of two lists, the result is a matrix of Venn diagrams and corresponding *p*-values that are calculated by the hypergeometric distribution. In the case of three lists, a cube is computed respectively. Then, a backtracking step is carried out similar to the backtracking in the dynamic programming solution of sequence alignments. For each position in the matrix (*i*,*j*) that contains the *p*-value for the first *i* and *j* elements, the matrix positions (*i*−1,*j*) and (*i*,*j*−1) are considered. Thereby, in each step it is decided whether an element of the first or the second list has to be added in order to come to the optimal *p*-value. In the case of three lists, the same applies for (*i*,*j*,*k*). In each case, the result of the algorithm is the sequence of items of the two or three sets that have to be added in order to find the Venn diagram with the most significant overlap. Here, the user has the choice either to compute the nominal *p*-values without adjustment, or to use either the Bonferroni or Benjamini-Hochberg adjustment. The standard choice of DynaVenn is to consider the Benjamini-Hochberg approach to control the false discovery rate (FDR). The formula used to compute the p-values (2 sets) is presented below:

$$ p\_value = 1 - \sum\limits_{l=1}^{|A[1:i] \cap B[1:j]| - 1} \frac{\binom{i}{l} \binom{|A \cup B | - i}{j-l}}{\binom{|A\cup B|}{j}}   $$

### Representation of results

**Fig. 1:**
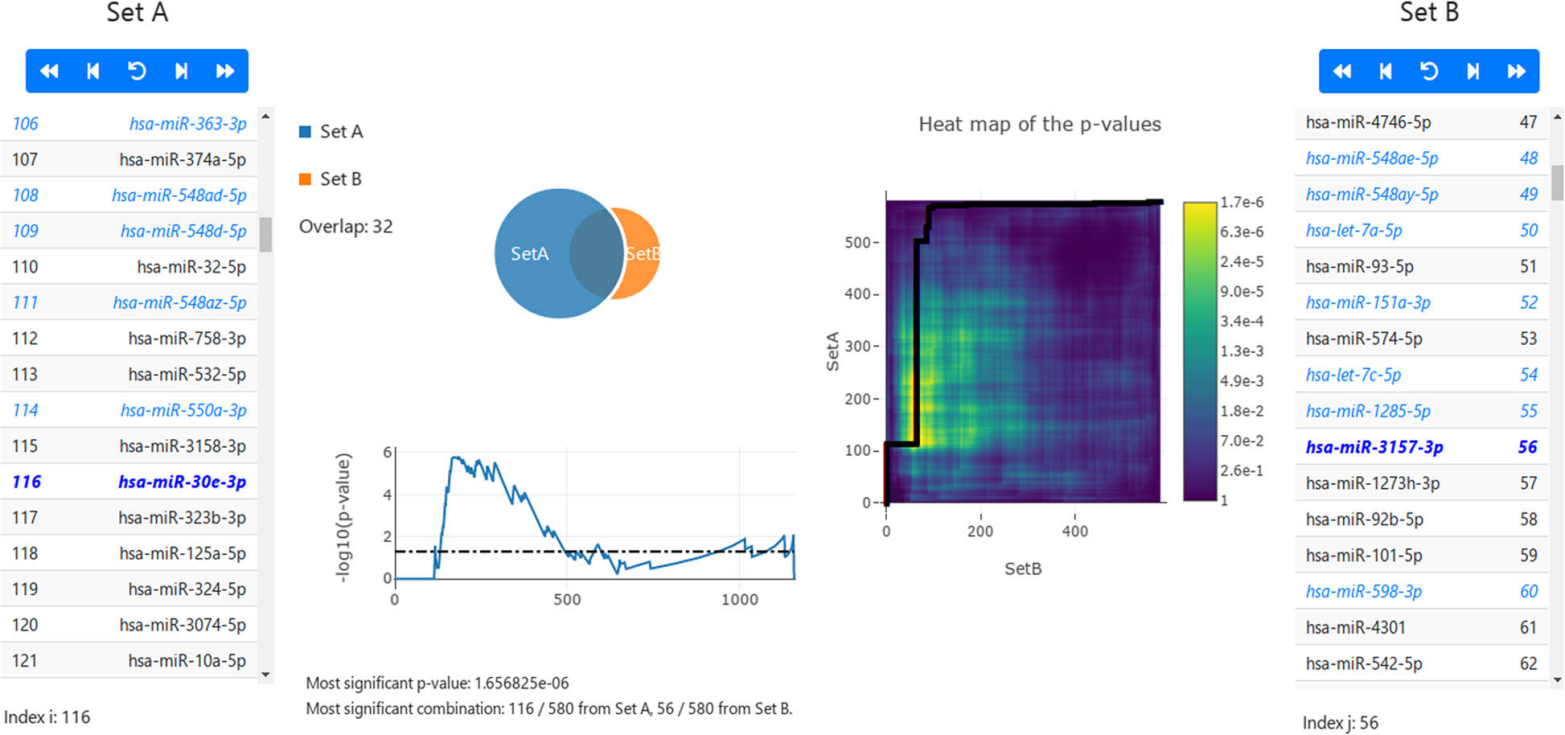
Results visualization of *DynaVenn*. Overview of the results using *DynaVenn* on the Alzheimer’s disease data set. The Venn diagram for the best combination of indices *i* and *j* is visualized, as well as the backtrace of p-values and the corresponding heat map

The results of the computation are visualized as area proportional Venn diagram in the central part of the results panel on the website (Fig. 1). Besides the diagram, the input lists are presented and the cut-off values selected by our algorithm are shown. Further, the elements that are contained in both lists are highlighted in blue. The user can run an animation: starting with an empty Venn diagram, the elements from both lists are step wise added according to the order selected by the backtracking algorithm until both lists have been completely added. Below the Venn diagram, the course of the *p*-value is presented. The best (lowest) *p*-value determines the best Venn diagram with the most significant overlap. The matrix containing the computed *p*-values can be visualized as a heat map.

### Implementation

The main algorithm of *DynaVenn* has been implemented in Python, including Celery and redis. The hypergeometric tests have been calculated using scipy, the adjustment for multiple testing is done with the statsmodels module. The visualizations have been created using venn.js and plotly.js. The results can be downloaded as JSON files for further offline analysis.

### Run time consideration

**Fig. 2:**
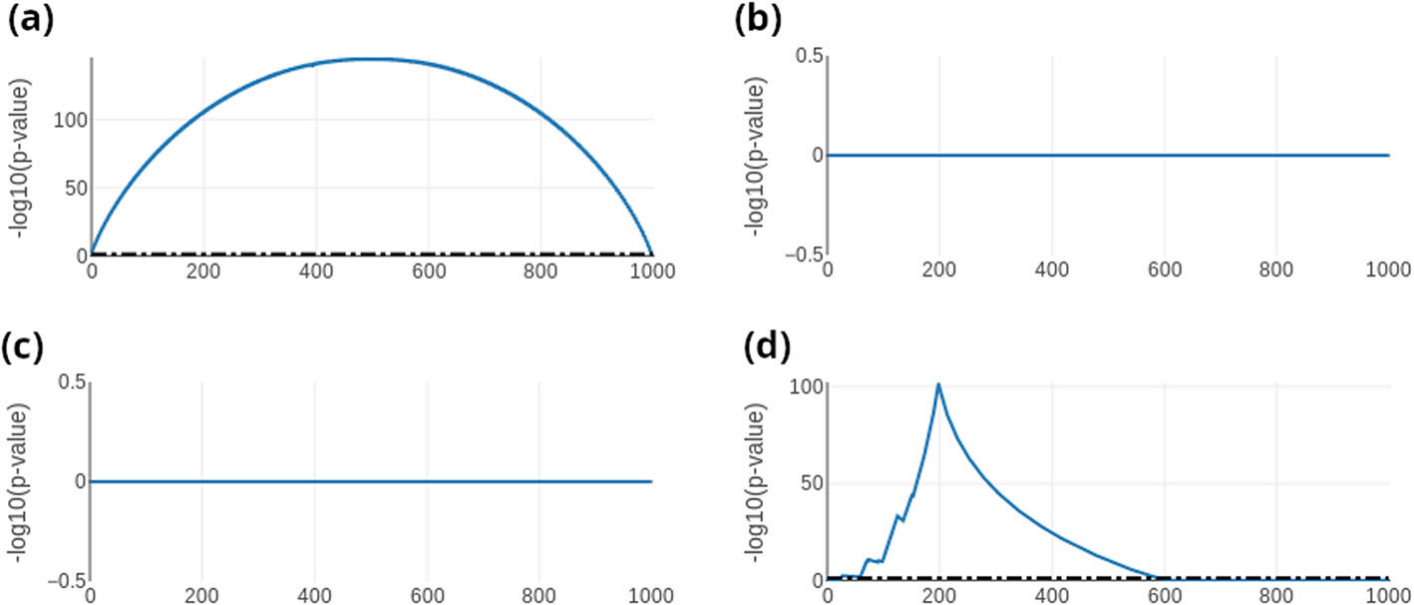
Toy examples. Four toy examples that demonstrate the ability and characteristics of the hypergeometric test in the context of gene list comparison. **a** Two identical lists. **b** The list and a randomly shuffled version of the list. **c** Complete list reversed. **d** The list with the head (20% reversed and the tail (80% reversed))

To understand the implication of the input size on the run time we increased random input sets in a step-wise manner and repeated the computations 10 times. The reported run times are averaged and the standard deviation is computed. Starting from input sets of size 10 over 20, 50, 100, 150 up to finally 1,000 items, the run time depending on the set size is presented in Fig. 2. As expected from the implementation, the run time increases quadratically with increasing set sizes, i.e. given list containing n items the asymptotic run time is $\mathcal {O}(n^{2})$. For typical input sets in the range of several hundred items, the execution time is still moderate an in the range of seconds, for 200 items in each list, DynaVenn e.g. requires 8 seconds to compute all 40,000 Venn diagrams, *p*-values and the backtracking. Since often also sets in the range of thousands will be explored an approximated time to result is interpolated and provided to the user. In case that a third list is used as an input, the run time becomes cubic, i.e. the asymptotic run time is $\mathcal {O}(n^{3})$. In this case it is not reasonable to upload lists with several thousand items.

## Results

### Selected extreme scenarios

To check the performance of the tool and to verify properties of the hypergeometric test and their effect on interpretability we created extreme cases. First, we used identical lists as input. Second, we flipped the both lists, i.e. the second list was the reverse as the first list. Third, we flipped the complete list. In a last example, we flip the first 20 percent and the last 80 percent of the lists each. These four scenarios are presented in Fig. 2a-d. For the identical sets (Fig. 2), the most significant overlap (in the case of 500 items the p-value is 1.5·10^−145^) is found to be in the middle of both lists after selecting 250 items from the first and 250 items from the second list, which is intuitive because of the symmetric nature of the hypergeometric distribution. For completely swapped lists, as well as in reversed lists (Fig. 2b-c) there is no overlap with p-value below 1. In the last case that is shown in Fig. 2d, where the top 20% and the bottom 80% of the second list are swapped compared to the original list, the most significant overlap is reached after selecting 200 markers (again, 500 markers are chosen as input set size), 100 from the first set and 100 from the second set. In this case, the *p*-value is 1.2·10^−102^.

### Application Alzheimer’s Disease

As biomedical use case we selected blood-borne microRNA Alzheimer’s Disease (AD) biomarkers. In a first study, we evaluated a cohort of American patients using Illumina Next Generation Sequencing (NGS) [8]. In a second study we followed a similar study protocol in Germany and applied basically the same experimental technique [9]. In the latter manuscript we state “In the initial cohort, we observed 203, in the validation cohort, 146 dysregulated miRNAs at a significance level of 0.05. With 68 miRNAs, the overlap was significant (P =.0003)”. This means that we selected all 203 nominally significant miRNAs from the first study performed in the US, all 146 nominally significant miRNAs from the validation study in Germany and computed 68 overlapping miRNAs that have been shown as a Venn diagram. Using the hypergeometric distribution, we computed a *p*-value of 0.0003 for this overlap. The two miRNA list, i.e. all miRNAs sorted by *p*-values in ascending order were used as input for *DynaVenn*. *DynaVenn* selected the top 116 miRNAs from the primary study, 56 from the second study, yielding an overlap of 32 miRNAs in both sets (Fig. 1). The FDR-adjusted p-value for this overlap was 1.66·10^−6^, 3 orders of magnitude below the original *p*-value (raw *p*-value: 7.7·10^−11^). In this case, *DynaVenn* supported to find a more significant overlap for the original and the validation set. The decreased *p*-value is thereby mostly due to a substantially lower number of miRNAs selected from the original data set.

### Application to over three lists

As described above, the run time of DynaVenn increases exponentially with the number of ordered lists to be compared. For *m* lists with *n* items the run time is $\mathcal {O}(n^{m})$. For comparing larger numbers of ordered lists heuristic solutions can be applied. The most straightforward way is to pick the most significant overlap between two ordered lists and use this as an input for further iterations. More sophisticated strategies such as computing and comparing all pair-wise combinations of two lists and step-wise joining those with the highest overlap resulted in very long run times of DynaVenn. In that we consider the maximum of three lists as one of the currently most relevant limitations of DynaVenn.

## Conclusions

Researchers have many options to compare sets or lists in terms of similarity. For lists, e.g. different metrics are available. One way that is frequently applied by researchers is to use Venn diagrams, a class of set diagrams. Here, it is assumed that the order of the items does not play a role. One case in biomedical applications is to compare items with *p*-values below a certain threshold in two experiments to get an understanding whether significant items in both lists overlap. With *DynaVenn* we developed a tool that does not rely on a fixed cut off value for two or three input lists but computes the optimal threshold and thus the most significant Venn diagram from the input lists. We demonstrate the improved performance on an Alzheimer’s Disease biomarker set but also consider extreme cases. When the lists are equal, a metric for the similarity of lists may be more appropriate since *DynaVenn* considers the middle of the lists as most significant overlap, which is correct given that it considers the lists as sets. In the third extreme case, where the top 20% of the list and the bottom 80% of the list are flipped, *DynaVenn* computes the perfect Venn diagram within few seconds.

Our tool reports well interpretable graphics and more over presents the results also as interactive simulation. A challenge is the run time. In case of two lists, the result is computed even for hundreds to thousands of items in seconds to minutes. For three sets, the run time increases in a cubic manner, restricting the input to several hundred but not thousands of items. There exist several ways to improve this, on an implementation side, parallelization may lead to a speed up of three orders of magnitude. Additionally, a pruning strategy may help to find areas in the matrix or cube, where significance values will exceed a certain threshold and have thus not to be considered. Further, also the extension to larger numbers of lists can be addressed in further versions of DynaVenn.

In sum, we provide an easy to use online tool that makes static Venn diagrams dynamic and serves many different applications in biomedical research and beyond.

## Availability and Requirements

Project name : DynaVenn Project home page : http://www.ccb.uni-saarland.de/dynavennOperating system(s) : Platform independent Programming language : Python, Javascript Other requirements : Tested with Safari, Firefox and Chrome browsers License : Proprietary Any restrictions to use by non-academics : The source code of the project can be made available for non-commercial use upon request.

## Data Availability

The data are publicly available [8–10]

## Abbreviations

AD: Alzheimer’s disease
ChIP-Seq: Chromatin ImmunoPrecipitation DNA-Sequencing (ChIP-Seq)
FDR: False discovery rate
miRNA: microRNA
NGS: Next Generation Sequencing
